# Information presentation through a head-worn display (“smart glasses”) has a smaller influence on the temporal structure of gait variability during dual-task gait compared to handheld displays (paper-based system and smartphone)

**DOI:** 10.1371/journal.pone.0195106

**Published:** 2018-04-09

**Authors:** Alireza Sedighi, Sophia M. Ulman, Maury A. Nussbaum

**Affiliations:** 1 Department of Industrial and Systems Engineering, Virginia Tech, Blacksburg, Virginia, United States of America; 2 School of Biomedical Engineering and Sciences, Virginia Tech, Blacksburg, Virginia, United States of America; University of Illinois at Urbana-Champaign, UNITED STATES

## Abstract

The need to complete multiple tasks concurrently is a common occurrence both daily life and in occupational activities, which can often include simultaneous cognitive and physical demands. As one example, there is increasing availability of head-worn display technologies that can be employed when a user is mobile (e.g., while walking). This new method of information presentation may, however, introduce risks of adverse outcomes such as a decrement to gait performance. The goal of this study was thus to quantify the effects of a head-worn display (i.e., smart glasses) on motor variability during gait and to compare these effects with those of other common information displays (i.e., smartphone and paper-based system). Twenty participants completed four walking conditions, as a single task and in three dual-task conditions (three information displays). In the dual-task conditions, the information display was used to present several cognitive tasks. Three different measures were used to quantify variability in gait parameters for each walking condition (using the cycle-to-cycle standard deviation, sample entropy, and the “goal-equivalent manifold” approach). Our results indicated that participants used less adaptable gait strategies in dual-task walking using the paper-based system and smartphone conditions compared with single-task walking. Gait performance, however, was less affected during dual-task walking with the smart glasses. We conclude that the risk of an adverse gait event (e.g., a fall) in head-down walking conditions (i.e., the paper-based system and smartphone conditions) were higher than in single-task walking, and that head-worn displays might help reduce the risk of such events during dual-task gait conditions.

## Introduction

Diverse activities impose simultaneous physical and cognitive demands [1]. Combat actions, use of a computer [2], and manufacturing tasks [3] are several examples of such *dual task* activities in the occupational domain. It remains unclear, however, whether such activities impose higher risks of adverse outcomes, such as injury, as existing evidence is somewhat mixed. Mental loads imposed along with physical demands have been suggested as increasing the risk of injuries through decreased muscle endurance, delayed muscle recovery [4], increased spinal loads [5], and gait variabilities [6]. However, other studies have found inconsistent results; for instance, adding mental loads to physical tasks has been found to have no or only slight effects on muscle activities [1, 7, 8], and even decreased activity in some cases [9]. It thus appears that different levels of physical and/or mental demands can lead to diverse outcomes [1].

Use of information displays is one example of a dual-task activity, and is of particular interest to us given the growing use among the general public and since various industries are interested in and have begun using these technologies. Modern information displays can enhance human interaction with environments and can augment human perception to perform tasks or detect risks. There are diverse information displays, and among these head-worn displays (HWDs, including “smart glasses”) have received recent attention, including for industrial applications, because of features including the ability to provide visual and audio information, to receive vocal commands, and to enable hands-free information exchange [10]. HWDs have been applied successfully in design, manufacturing, assembly, and logistics industries [11–15]. Findings of these previous studies imply that using a HWD can increase a worker’s performance, yet the broader effects of HWD use, as an additional source of mental workload, has not been fully described. Therefore, we suggest there is a current need for additional investigation of the potential impacts of HWDs on performance, particular primary task performance, since there may be unintended or unexpected adverse outcomes.

Smartphones, auditory devices [11], and paper-based methods are among the more widely used methods in industry to provide instructions and/or communicate with workers. Each of these methods is likely to have different impacts on workers’ performance, since the required attentional demands vary. Therefore, it is of interest to investigate whether the effects of HWD use (as a mental load) on performance are distinct when compared to these other methods. He et al. [16] addressed this issue in the context of a driving task, and their results suggested that a HWD is safer than a smartphone. However, no prior work addressed this question in the context of other common activities.

Walking is a routine and common human activity, and for many industries it is one of the most common physical activities performed [17]. While apparently a simple activity, walking is actually a complex task that is highly dependent on cognitive resources and the sensorimotor system [18]. When humans perform both a cognitive task and walking simultaneously, the central nervous system (CNS) needs to allocate limited attentional resources between both tasks to complete the dual-task activity successfully. As such, variability in gait is used commonly as a proxy to indicate a decline in resources allocated towards performing the walking task [18, 19]; such a decline, in turn, can be used to infer potential risks, such as a loss of balance or a fall. For example, several studies have investigated the relationship between differing mental loads and gait performance [20–26]. Results of these studies suggest that changes in the variability of gait parameters (as indicators of walking performance)–specifically increases or decreases in gait variability compared to single-task walking–are evident only when the level of mental load exceeds specific thresholds. We thus suggest that prior to using new devices, such as HWDs, that impose new and/or additional cognitive loads during walking tasks, it is essential to evaluate their effects and in particular to assess whether use of such devices might increase risk.

Due to the large number of kinematic degrees-of-freedom (DOFs) in the human body [27, 28], abundant solutions exist for overcoming or adapting to the effects of cognitive loads on the performance of certain complex tasks, such as walking, and which lead to inherent variations in body movement [29]. While these variations once were thought to be sensorimotor noise [30, 31], they have more recently been identified as an essential movement characteristic [32], and are termed motor variability (MV). In the other words, MV includes any variations in the spatiotemporal distributions of joint movements, inter-joint coordination patterns, muscle activities, and gait parameters. One question this study poses is whether the CNS benefits from MV to successfully maintain walking performance in the presence of different additional sources of mental load, and whether the specific source of information presentation is influential.

Prior to exploring this question, however, an appropriate method for quantifying MV must first be determined. In the field of motor control, this remains challenging due to the variety of methods that currently exist for measuring movement variations. Three different classes of approaches have been typically used in assessing MV: 1) linear methods, 2) methods stemming from chaos theory, and 3) methods based on the numerous DOFs within the human body [33]. The first class, and also the traditional approach, includes linear methods based on descriptive statistics (e.g., standard deviation), and has been implemented for both discrete and continuous measures. The second class, inspired by chaos theory, incorporates nonlinear methods that have recently gained traction in the field of human movement (e.g., sample entropy and Lyapunov exponent). The final class considers the abundant DOFs accessible to execute a repetitive task, which has been termed “equifinality” [34]. Several methods have been introduced to quantify MV based on “equifinality”, including the uncontrolled manifold [28, 35], tolerance noise covariation [36], minimum intervention principle [37], and goal equivalent manifold (GEM) [34]. Among the methods in the noted three classes, the GEM method is the only approach that can be employed to *simultaneously* quantify both variability in magnitude and the temporal structure of variations [38]. The GEM method has recently been implemented successfully in studies of diverse activities such as lifting [39], trunk flexion/extension [40], reaching [41], and walking [18, 42–44].

As previously discussed, we consider that it is important to understand if and how using a HWD, such as smart glasses, influences gait variability, and whether the risk of using such a device differs from that involved when using more traditional methods of information presentation (smartphones and paper-based systems). Additionally, evidence is needed to help identify the most appropriate method for quantifying MV, to best detect the effects of various information displays on gait performance. To address these needs, an experiment was completed to test three hypotheses. First, that an increase in gait variability will occur as an adaptive response when using an information display (smart glasses, smartphone, or paper-based system) while walking. Second, that gait performance is less adversely influenced when participants use smart glasses compared to using either a smartphone or paper-based system. Third, that diverse measures of MV have varying levels of sensitivity to changes induced by different dual-task conditions (information displays) in the context of gait.

## Method

### Participants

Ethics Statement: Prior to any data collection, all participants provided informed consent by reviewing and signing a consent form that described the aims and procedures of the study. The study procedures, including the consent form, were approved by the Virginia Tech Institutional Review Board (IRB #16–420).

A total of 10 females and 10 males completed the current experiment (Table 1). Participants were recruited from among the local student population, and needed to meet several criteria. Our initial target population was primarily healthy young people, considered most likely in the near future to use HWDs on a regular basis. As such, and based on previous work [16, 18], we limited participation to those who were 18–35 years old and who had no self-reported current or recent history of musculoskeletal disorders or neurologic problems. Participants were also required to have normal vision, or corrected vision with contact lenses [16], which was confirmed using a Snellen eye chart. We excluded participants who wore eyeglasses since it was not feasible to use smart glasses and eyeglasses at the same time. Finally, participants were required to have a smartphone or experience in using smartphones, and to be fluent in English (to be able to complete several aspects of the experiment).

**Table 1:** Mean (SD) information of the study participants.

	Age (years)	Body mass (kg)	Stature (cm)
Males	23.9 (3.2)	74.8 (13.0)	176.5 (12.6)
Females	22.3 (2.5)	66.2 (13.5)	164.5 (7.6)

### Experimental procedures

**Fig 1:**
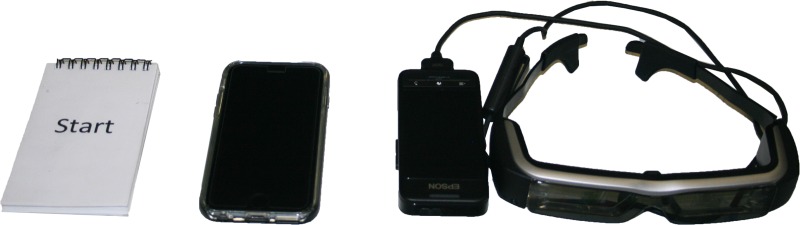
The different information displays. Left: paper-based system, middle: smartphone, Right: smart glasses.

Participants completed a preliminary session and then an experimental session on a subsequent day. In the preliminary session, all experimental procedures were explained, and several anthropometric measures were obtained. Then, participant’s preferred walking speed (PWS) was determined using a treadmill (h/p/cosmos gaitway® II S, KISTLER and h/p/cosmos, Nussdorf-Traunstein, Germany) using procedures based on the protocol introduced by Jordan et al. [45]. Initially, the treadmill speed was set at a low value, which was then incremented in steps of 0.1 km/h until a participant indicated that it was their preferred speed. In the next stage, the protocol started with a speed that was 1.5 km/h higher than the reported PWS, and was decremented by 0.1 km/h until the participant indicated that the speed was their preferred one. This procedure was repeated until the difference between speeds identified in the two approaches was < 0.4 km/h. We also provided sufficient time for participants to familiarize themselves with three different information displays (Fig 1); a paper-based system; a smartphone (iPhone 6S, Apple, Cupertino, CA); and smart glasses (Moverio BT-200, Epson). The screen/page and font size were the same for all three displays. For the smart glasses, the screen positioning was not adjustable. However, it was optimized prior to data collection by adjusting the mapping surface, which was a black sheet hung from the ceiling in front of the treadmill. The preliminary session ended with participants walking on the treadmill while performing several cognitive tasks using the three display methods. These cognitive tasks were a shorter version of the tasks used during the actual experiment, as described below.

**Fig 2:**
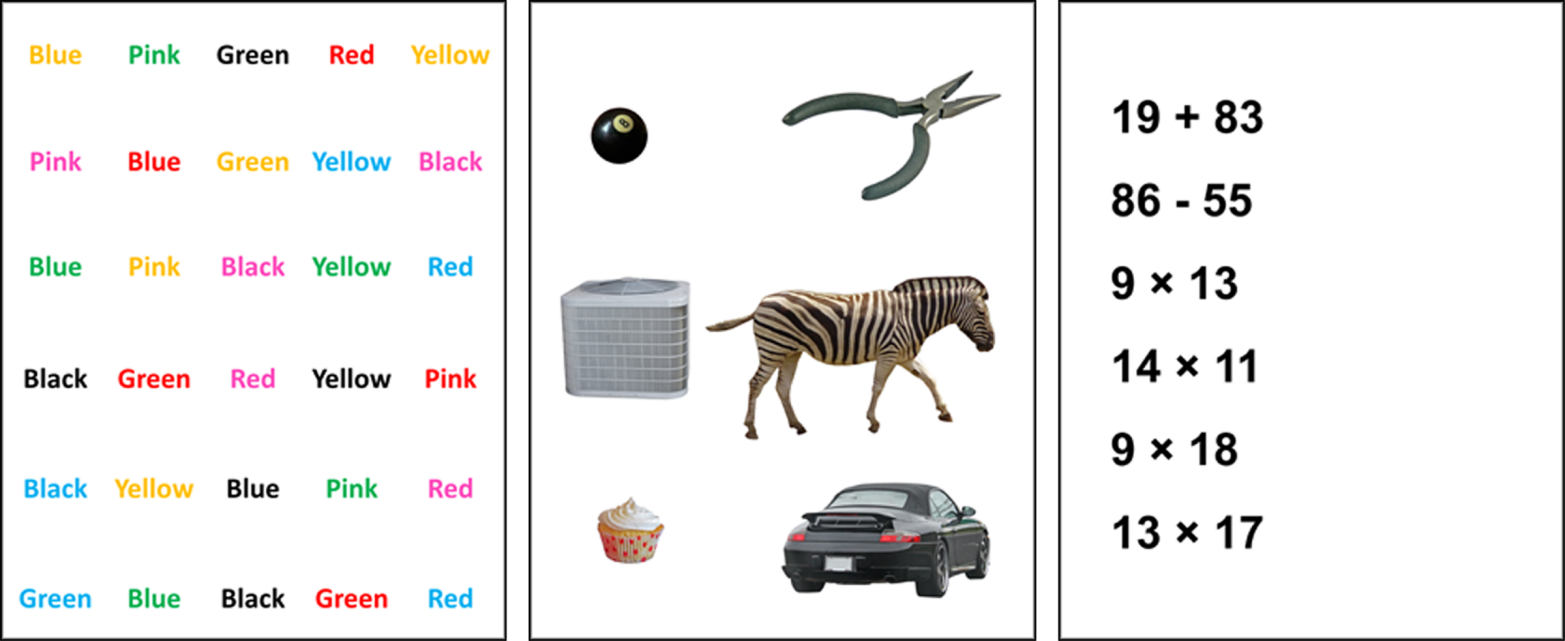
Sample illustrations of the three cognitive tasks. Left: Stroop test, Middle: Categorization task, Right: Mental arithmetic task.

In each of the experimental conditions described subsequently, participants were asked to perform a set of activities that involved three different cognitive tasks (Fig 2): 1) the Stroop test [46]; 2) object categorization; and 2) mental arithmetic. These three tasks were chosen to increase attentional demands in different ways and to simulate common activities that might be performed when using information technologies. For each cognitive task, participants were instructed to give as many responses as they could, and to be as accurate as possible while maintaining a comfortable pace; 5-second breaks were provided between each task. Pilot work was conducted to determine the duration of exposure periods for each task. Exposure periods during the experiment were chosen such that a majority of participants would not complete the tasks within the given periods (since otherwise there would be a need to repeat informational material).

The Stroop test involved a series of color words printed in differing colors (e.g., the word “Red” was printed in blue). For this task, the participant was asked to recite the printed colors aloud and in order, as they read a displayed list that was visible for 16 seconds. In each experimental condition, the Stroop test was given five times; each time, 30 color words were randomly chosen and positioned on the list. Randomization was constrained to six colors: blue, red, green, yellow, black, and pink. Therefore, for the 30 color words, each color appeared five times each as a word and color. For example, the word “Red” appeared in the list five times and there were five words that were colored red.

The categorizing task was inspired from the Boston Naming Test [47], an assessment tool that measures word retrieval ability [48]. To implement this task, pictures were randomly selected from a database [49] that normalized the pictures based on category. The participant was asked to state aloud the category (i.e., animal, tool, electronic, game, food, and vehicle) associated with each picture displayed. For each experimental condition, this task was given four times; each time, three slides were presented for 18 seconds with six pictures on each slide. All six categories were represented on each slide in a random order.

For the third cognitive task, a series of six arithmetic problems were presented, in increasing difficulty. The first and second problems required the addition and subtraction of two-digit numbers, respectively, with the numbers selected randomly. The remaining four problems were multiplication; to gradually increase difficulty, the first two multiplication problems involved multiplying a single digit and a two-digit number, and the final two problems involved multiplying two two-digit numbers (all numbers were randomly selected). For each experimental condition, this task (set of six problems) was presented four times, and each time participants recited the solutions aloud while the problems were displayed for 20 seconds.

**Fig 3:**
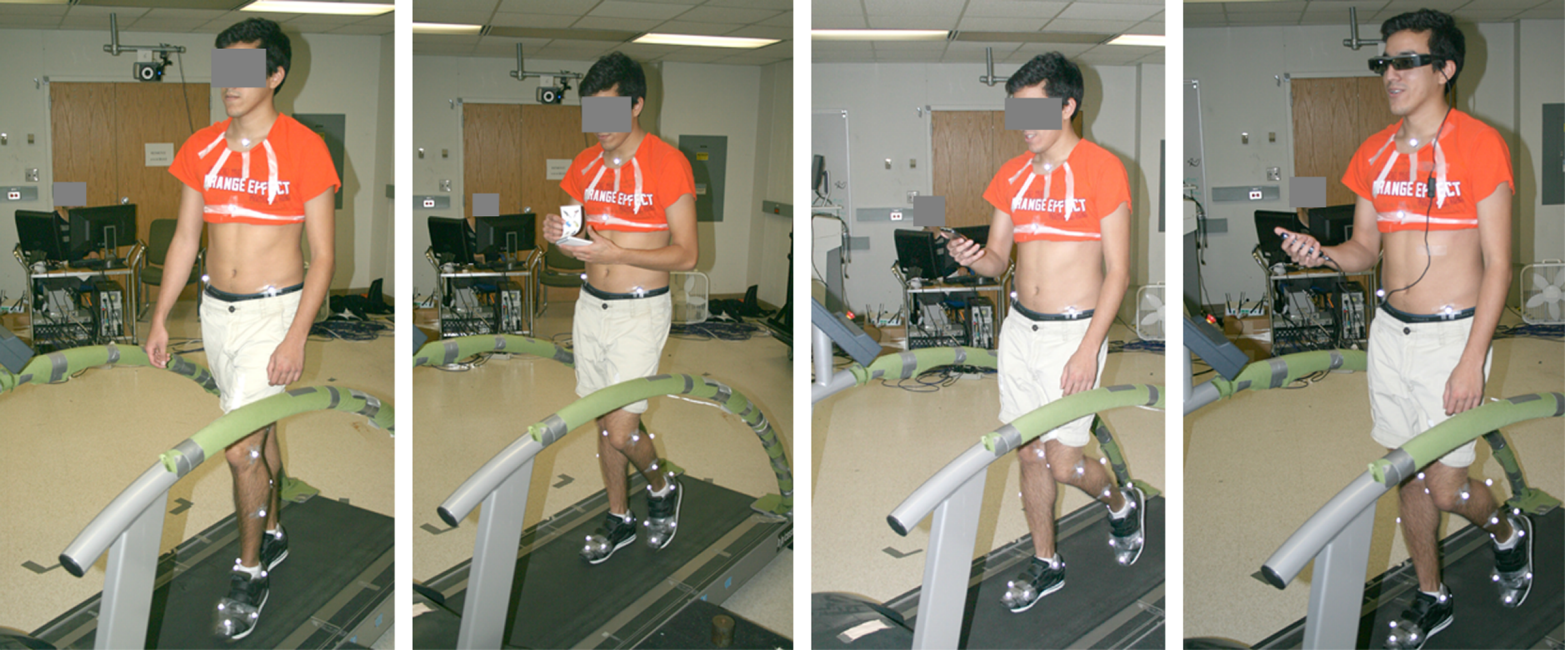
Illustration of the different walking condition. From left to right: single-task walking; dual-task walking using the paper based system; dual-task walking using the smartphone; dual-task walking using the smart glasses. The individual in this manuscript has given written informed consent (as outlined in PLOS consent form) to publish these case details.

In the experimental session, each participant completed one training trial and four walking trials. In the training trial, participants were asked to sit on a chair and perform the three cognitive tasks as described above (total duration ~ 5 minutes). During this trial, we ensured that participants had no difficulty with color identification. After this, they completed four 5-minute walking trials on the treadmill, at their PWS, in each of following display conditions (Fig 3): 1) single-task walking (ST), with no cognitive tasks; 2) dual-task (DT) walking while completing the cognitive tasks using the paper-based system (DT-paper); 3) DT walking while completing the cognitive tasks using the smartphone (DT-phone); and, 4) DT walking while completing the cognitive tasks using the smart glasses (DT-glass). The duration of each walking condition was set to 5 minutes to ensure that sufficient data points were available for calculating variability measures (see below). Similar to a previous study [18], PWS was determined here for a single-task walking during the preliminary session, and we used the same speed for all of the four walking conditions tested. The participants also were asked to look forward in the ST and DT-glass conditions, whereas in the two other conditions (i.e., DT-paper and DT-glass) they had to look down. In all conditions, a black sheet hung from the ceiling in front of the treadmill to provide a consistent background for the participants, and to act, as noted earlier, as a mapping surface for the smart glasses. Confounding effects related to walking trial order were minimized by counterbalancing the order of the four display conditions, using five 4 × 4 Balanced Latin Squares. However, the order of the cognitive tasks remained consistent with the following order: Stroop test, categorization, and mental arithmetic. Additionally, each experimental condition required the participant to hold an object, specifically the paper, smartphone, and smart glasses’ controller. Confounding effects related to hand posture were minimized by asking the participant to hold the object in the same hand and position throughout all conditions; self-determined comfortable hand and posture used were determined initially by participants, prior to data collection.

### Data collection and processing

Reflective markers were used to capture 3D segmental kinematics during the walking tasks. These markers were placed over anatomical landmarks on the participants’ lower limbs [18] and trunk, and a 7-camera system (Vicon Motion System, CA, USA) tracked the marker positions at 100 Hz. All kinematic data were processed using Vicon Nexus software and Matlab (MathWorks, Inc., Natick, MA).

After finishing each experimental condition, perceived mental workload was assessed using the NASA task load index (TLX) [50]. This assessment tool considers six subscales addressing mental, physical, and temporal demand, along with performance, effort, and frustration. Ratings for each are given on a 100-point scale and are combined to calculate the NASA task load index. Participants also completed two questionnaires (Table 2), each using five-point scales; a usability questionnaire [51] (1 = strongly disagree, 3 = moderately agree, and 5 = strongly agree) and a questionnaire related to eye strain and discomfort [52] (1 = none, 3 = moderate, and 5 = very severe). After completing all four experimental conditions, participants were asked to rank the three different display methods (paper, smartphone, and smart glasses) in order of preference (1 = most preferred, 2 = second most preferred, 3 = least preferred), to explain their ranking, and to list perceived advantages and disadvantages for each display method.

**Table 2:** Usability metrics and associated questionnaire items.

***Ease of Use***
1. It was useful for completing the task
2. It was simple to use
3. I was satisfied with it
4. It worked the way I wanted it to work
***Discomfort***
5. General discomfort
6. Headache
7. Eye Strain
8. Nausea
9. Difficulty concentrating
10. Blurred vision
11. Imbalance/Disorientation

Several measures of gait MV were derived to determine the effects of the different display types. Within each condition (i.e., 5 minutes of treadmill walking), we first calculated basic gait parameters of stride length (*L*_*n*_), stride time (*T*_*n*_), and stride speed (*S*_*n*_) for each stride *n*, as well as step length; these were obtained using raw (unfiltered) kinematic data [43]. To calculate these gait parameters, we identified the times of heel strike using a common technique for treadmill walking [53]; heel contacts were identified at the maximum distance between the hip and heel markers in the anterior-posterior direction. We defined step lengths by calculating the distance between two heel markers at each heel contact event, when both feet were on the treadmill. We then added two successive step lengths to calculate stride length (*L*_*n*_) [44]. Stride time (*T*_*n*_) was determined as the duration between two consequetive heel contact events for the right foot. Lastly, stride speed was defined as stride length per stride time (*S*_*n*_ = *L*_*n*_/*T*_*n*_). For consistency here, and similar to previous studies [18, 43], we analyzed a fixed number of strides, which was determined based on the shortest stride count observed across all participants and conditions (i.e., 241).

Variations in these gait parameters were quantified using a method from each of the three classes previously described (i.e., linear, nonlinear, and equifinality). First, Cycle-to-cycle SDs (σ) of the gait parameters (i.e., stride length, stride time, and stride speed) were computed as a representative linear method [43]. Second, and similar to Yentes et al. [54], sample entropy (SaEn; developed by Richman and Moorman [55]) was used as a nonlinear method to quantify variations in *L*_*n*_, *T*_*n*_, and *S*_*n*_. SaEn was used here to measure the complexity of a time series, *X(i)* = *X(1)*, *X(2)*, *…*, *X(N)*, and can be calculated as follow:

$$SaEn(m,n,N)=-\text{ln}(\phi^{m+1}(r)/\phi^{m}(r))$$ (1)

in which *ϕ*^*m*^*(r)* is the mean of *C*^*m*^_*i*_ = (number of *X(j)* such that Chebyshev distance between *X(i)* and *X(j)* is less than *r*), and *N* is the total number of data points. As Yentes et al. [54] suggested, we explored different values of *m* (i.e., 2,3, and 4) and *r* (i.e., 0.05,0.1,0.15, 0.2,0.25, and 0.3 × SD of the time series). We only report results for *m* = 3 and *r* = 0.25 × SD, since it revealed more information (as discussed subsequently).

Third, the GEM framework was chosen from among available methods based on equifinality. Since participants walked on the treadmill at their PWS, it can be assumed the primary task goal was maintaining constant speed (*v*) at each stride. This can be formulated as *<L*_*n*_*/T*_*n*_*>*_*n*_ = *v*, in which *<·>*_*n*_ is an averaging function over *n* strides [44]. For a speed-based GEM analysis, all combinations of *L*_*n*_ and *T*_*n*_ that can satisfy the goal function (*L*_*n*_/*T*_*n*_ = *v*) describe the GEM. According to Dingwell and Cusumano (44), after normalizing (*L*_*n*_) and time (*T*_*n*_) to their SD, the magnitude of variability in the GEM direction (*δ*_*T*_), and in the direction perpendicular to the GEM (*δ*_*P*_), can be calculated as follows for each stride:

$$\left[\begin{array}{c} \delta_{T}\\ \delta_{P} \end{array}\right]=\frac{1}{\sqrt{1+v^{2}}}\left[\begin{array}{cc} 1 & v\\ -v & 1 \end{array}\right]\left[\begin{array}{c} L_{n}\\ L_{n}^{'} \end{array}\right]$$ (2)

For this equation *T*_*n*_*΄* is equivalent to *T*_*n*_ ˗ T^*^, and *L*_*n*_*΄* is *L*_*n*_ ˗ L^*^. Furthermore, (T^*^, L^*^) is the preferred operating point, at which T^*^ = *<T*_*n*_*>*_*n*_ and L^*^ = v T^*^. It should be noted that the goal of the task is not affected by variations in the GEM direction (*δ*_*T*_), while the goal is deteriorated by variations in the other, perpendicular direction (*δ*_*P*_) [44]. The SD of variations in both the GEM direction (*δ*_*T*_) and the perpendicular direction (*δ*_*P*_) were calculated to investigate the structure of variability that reflected the average behavior of the system [44]. We were also interested in studying the temporal structure of the time series (i.e., *δ*_*T*_ and *δ*_*P*_) to quantify stride-to-stride variabilities. For this, Detrended Fluctuation Analysis (DFA; see Pend et al. [56]) was used to compute the scaling exponent (*α*) of the time series [44]. The derived value of *α* indicates whether a time series is persistent (*α* > 0.5) or anti-persistent (*α* < 0.5). If anti-persistent, this would indicate that participants adjusted the time series frequently to maintain the goal. Alternatively, persistent correlations would indicate non-frequent adjustments [44].

From each cognitive task, percentages of completed responses were determined. (Due to the similarity between percent correct and percent completed, only the latter measure was assessed). These results reflected the level of performance in each condition, and therefore were interpreted as indicating the amount of allocated resources. Mean NASA-TLX ratings were obtained using an unweighted method [57] to assess the overall level of perceived workload in each condition. Mean responses from the usability and eye strain questionnaires were also determined to assess ease of use and discomfort, respectively (two data points were missing for one participant). Finally, rankings were compiled and all open-ended question responses were summarized.

## Statistical analyses

Separate analysis of variance (ANOVA: REML method) models were used to investigate the effects of the four different display conditions (DC: three displays + none) and gender (G) on each of the measures of gait variability (i.e., cycle-to-cycle SD, SaEn, and GEM-related measures). Preliminary analyses indicate that the order of exposure to the four display conditions did not have significant or substantial effects, so order was not included in the final ANOVA models. Another set of ANOVA models was used to evaluate how the three displays (D) and gender (G) affected cognitive performance (percentages of complete responses). In the latter ANOVA models, order effects were significant for responses to the Stroop test and ranking, and these effects were included in the final models. All summary results are reported as least square means (95% confidence intervals). Where relevant, paired comparisons were done using the Tukey HSD method [58], and interaction effects were explored using simple-effects testing. Parametric model assumptions were assessed, and in several cases (i.e., SaEn of all variables, cycle-to-cycle of stride time and stride speed, and mean of ease of use and discomfort) data transformations (e.g., log, square, and reciprocal transformations) were used to obtain normally distributed model residuals. *P*-values < 0.05 were considered statistically significant, and the sensitivity of dependent measures with respect to DC, D, and G were assessed by calculating effect sizes (i.e., partial eta-squared = *η*_*p*_^*2*^). We interpreted effect sizes qualitatively using Cohen’s criteria [59] (i.e., *η*_*p*_^*2*^ *>* 0.14: large effect, 0.01 < *η*_*p*_^*2*^ < 0.06: moderate effect, and *η*_*p*_^*2*^ < 0.01: small effect). Comparisons of effect sizes were done to determine the most appropriate method for distinguishing the effects of different information displays on gait parameters.

## Results

### Linear measures

**Fig 4:**
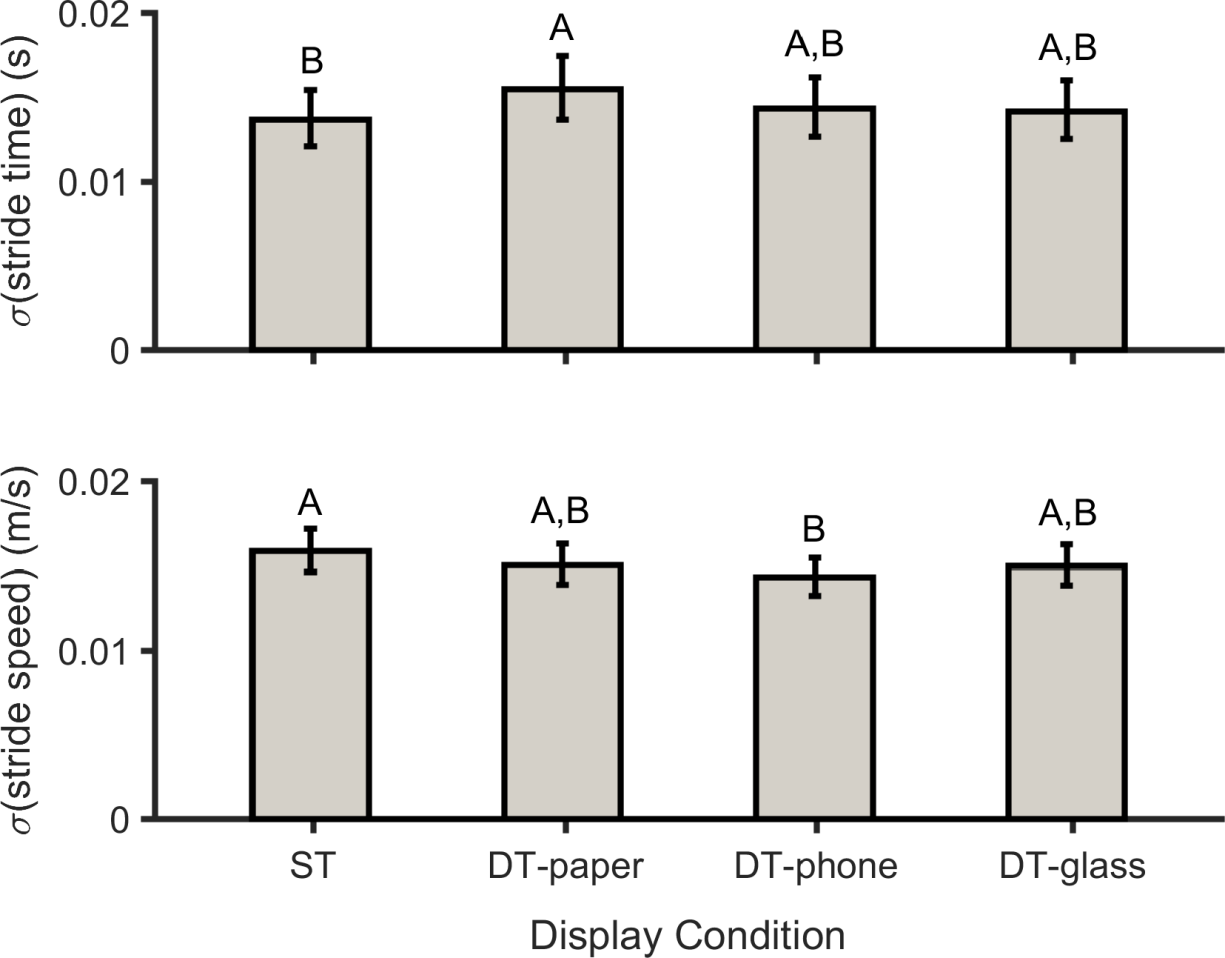
Cycle-to-cycle standard deviation (σ) of stride time (*p* = 0.008 and *η*_*p*_^*2*^ = 0.195; top) and stride speed (*p* = 0.006 and *η*_*p*_^*2*^ = 0.204; bottom) for single-task walking (ST), and for dual-task walking while using the paper-based system (DT-paper), smartphone (DT-phone), and smart glasses (DT-glass). The number of participants was 20. Values in conditions not sharing same letters are significantly different.

There were significant main effects of DC on the cycle-to-cycle SD of both stride time and stride speed (Table 3). Stride time variations increased significantly (*p* = 0.005) when participants used the paper-based system compared to the baseline (i.e., single task) condition. However, when participants used the two other displays (i.e., smartphone and smart glass), stride time variability was not noticeably affected (*p* = 0.529 and *p* = 0.719 respectively; Fig 4, top). Walking speed variations decreased significantly when using the smartphone compared to the single-task condition (*p* = 0.003), while these variations decreased slightly in the two other conditions (Fig 4, bottom).

**Table 3:** Summary of ANOVA results related to cycle-to-cycle SD (*σ*) outcomes for 20 participants. Both *p* values and effect sizes (*η*_*p*_^*2*^) are given for the main and interaction effects of different display conditions (DC) and gender (G), for the SD of stride length, stride time, and stride speed. Significant effects are highlighted using bold font.

		DC	G	DC×G
*σ* (Stride Length)	*p(η*_*p*_^*2*^*)*	0.122 (0.101)	0.867 (0.004)	0.432 (0.049)
*σ* (Stride time)	*p(η*_*p*_^*2*^*)*	**0.008 (0.195)**	0.819 (0.019)	0.450 (0.047)
*σ* (Stride speed)	*p(η*_*p*_^*2*^*)*	**0.006 (0.204)**	0.441 (0.127)	0.196 (0.082)

### SaEn results

**Fig 5:**
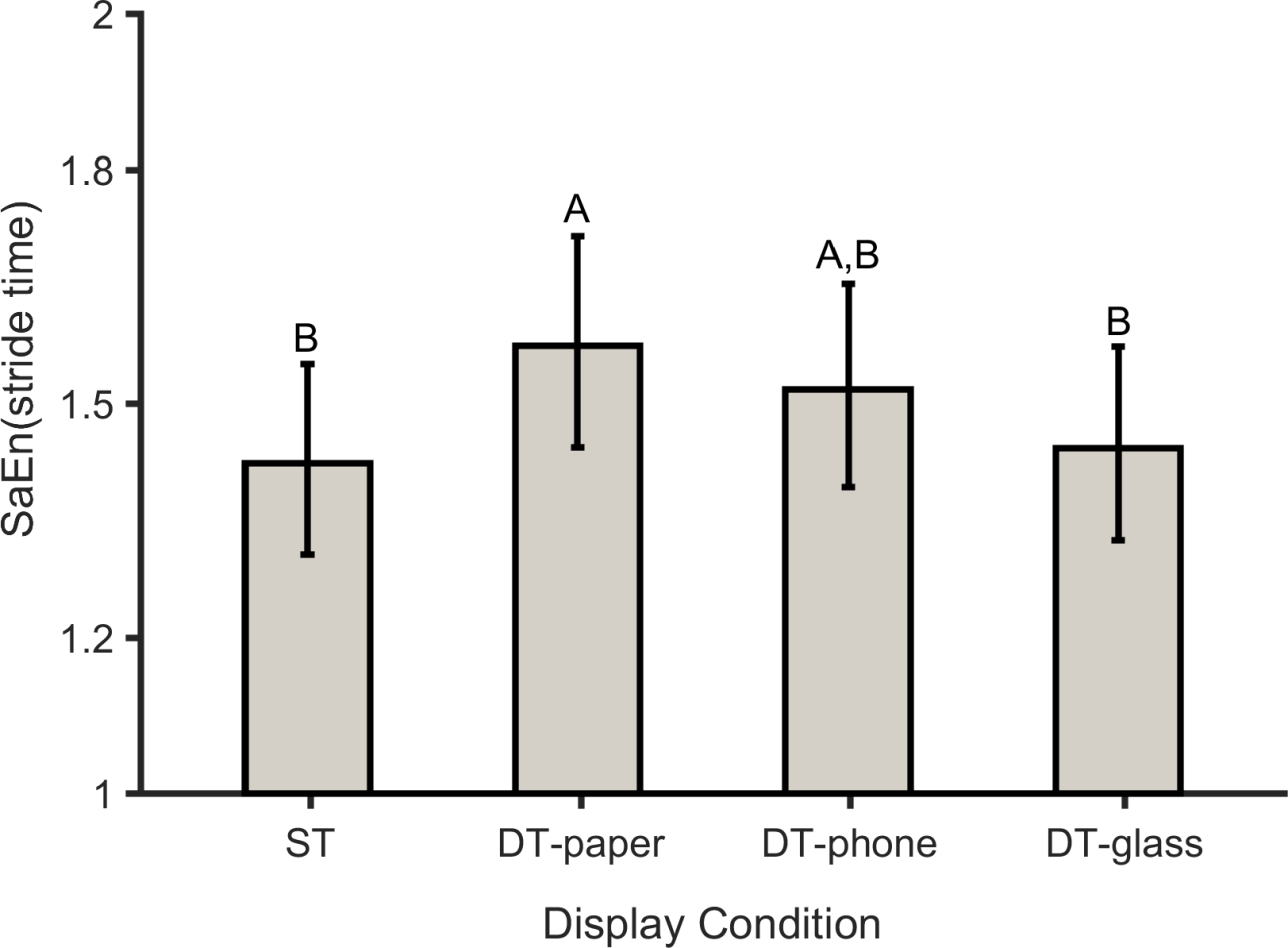
SaEn of stride time (*p* = 0.002 and *ηp2* = 0.244) for single-task walking (ST), and for dual-task walking while using the paper-based system (DT-paper), smartphone (DT-phone), and smart glasses (DT-glass). The number of participants was 20. Values in conditions not sharing same letters are significantly different.

For the nonlinear measure, there was a significant main effect of DC on the SaEn of stride time (Table 4, Fig 5). Compared with the ST condition, SaEn (stride time) was significantly (*p* = 0.003) higher in the DT-paper; it was also higher in the DT-phone condition, though this difference only approached significance (*p* = 0.096). In contrast, SaEn in the ST and DT-glass conditions were similar (*p* = 0.957). The gender × display condition interaction effect on SaEn (Stride Length) approached significance, with a medium effect size (Table 4). Simple effects testing indicated that SaEn (Stride Length) significantly decreased for females in the DT-paper and DT-phone conditions compared to the ST, but that values for the males were similar in all display conditions. In addition, SaEn (Stride Length) for females was significantly (*p* = 0.008) higher in the ST condition compared with males; it was also slightly higher in the DT-paper condition (*p* = 0.21). Values of SaEn of stride length were, however, similar for females and males in both the DT-phone and DT-glass conditions.

**Table 4:** Summary of ANOVA results related to the SaEn outcomes for 20 participants. Both *p* values and effect sizes (*η*_*p*_^*2*^) are given for the main and interaction effects of different display conditions (DC) and gender (G) for SD of stride length, stride time, and stride speed. Significant effects are highlighted using bold font and effects approaching significance are italicized.

		DC	G	DC×G
SaEn (Stride Length)	*p(η*_*p*_^*2*^*)*	0.271 (0.069)	0.561 (0.003)	*0*.*081 (0*.*116)*
SaEn (Stride time)	*p(η*_*p*_^*2*^*)*	**0.002 (0.244)**	0.604 (0.076)	0.378 (0.055)
SaEn (Stride speed)	*p(η*_*p*_^*2*^*)*	0.720 (0.024)	0.79 (0.001)	0.451 (0.047)

### GEM-based outcomes

**Fig 6:**
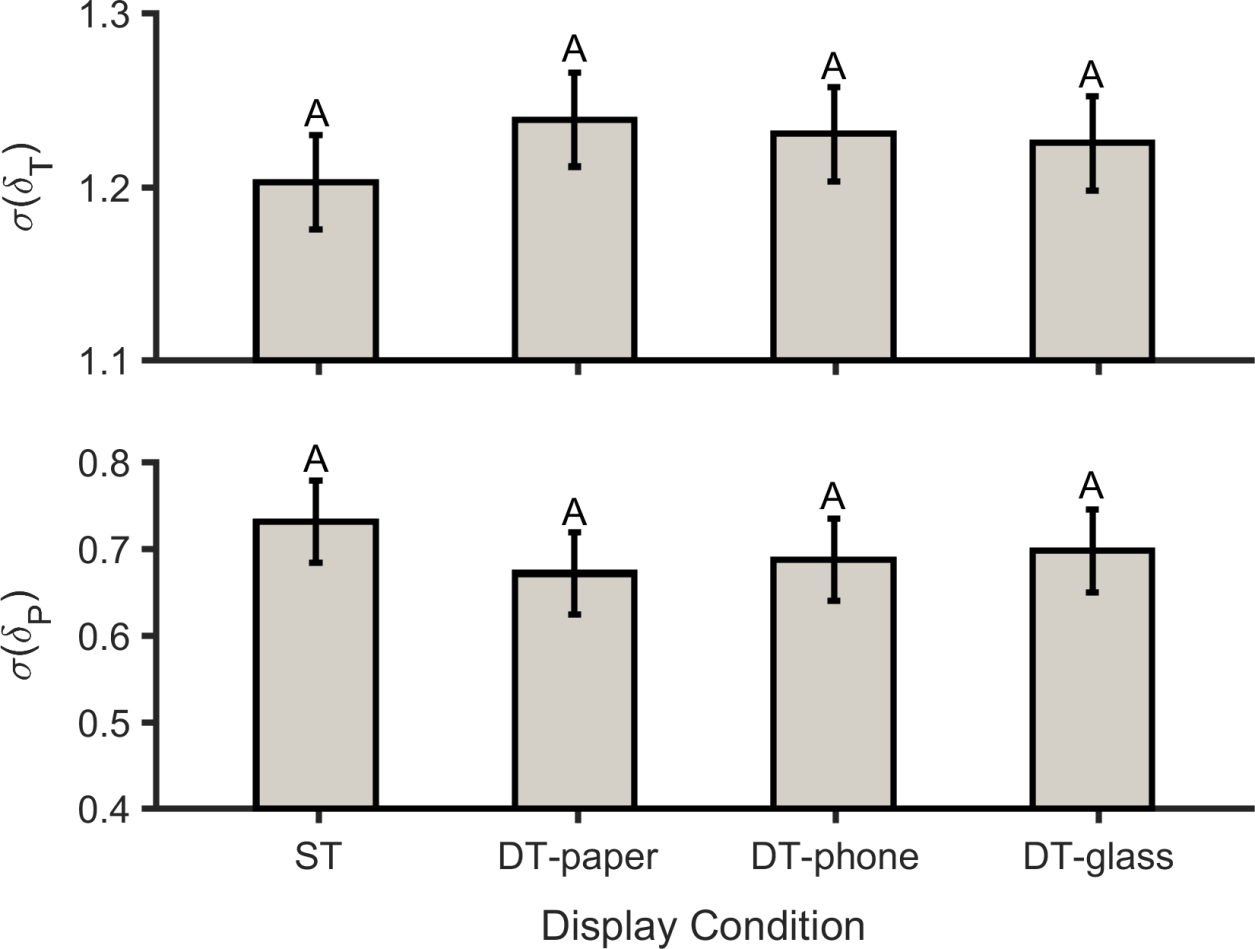
Magnitude of variability in the GEM direction (*p* = 0.071 and *ηp2* = 0.121; top) and in the direction perpendicular to the GEM (*p* = 0.108 and *ηp2* = 0.105; bottom), for single walking task (ST), dual walking task while using the paper-based system (DT-paper), dual walking task while using the smartphone (DT-phone), and dual walking task while using the smart glass (DT-glass). The number of participants was 20. Values in conditions not sharing same letters are significantly different.

**Fig 7:**
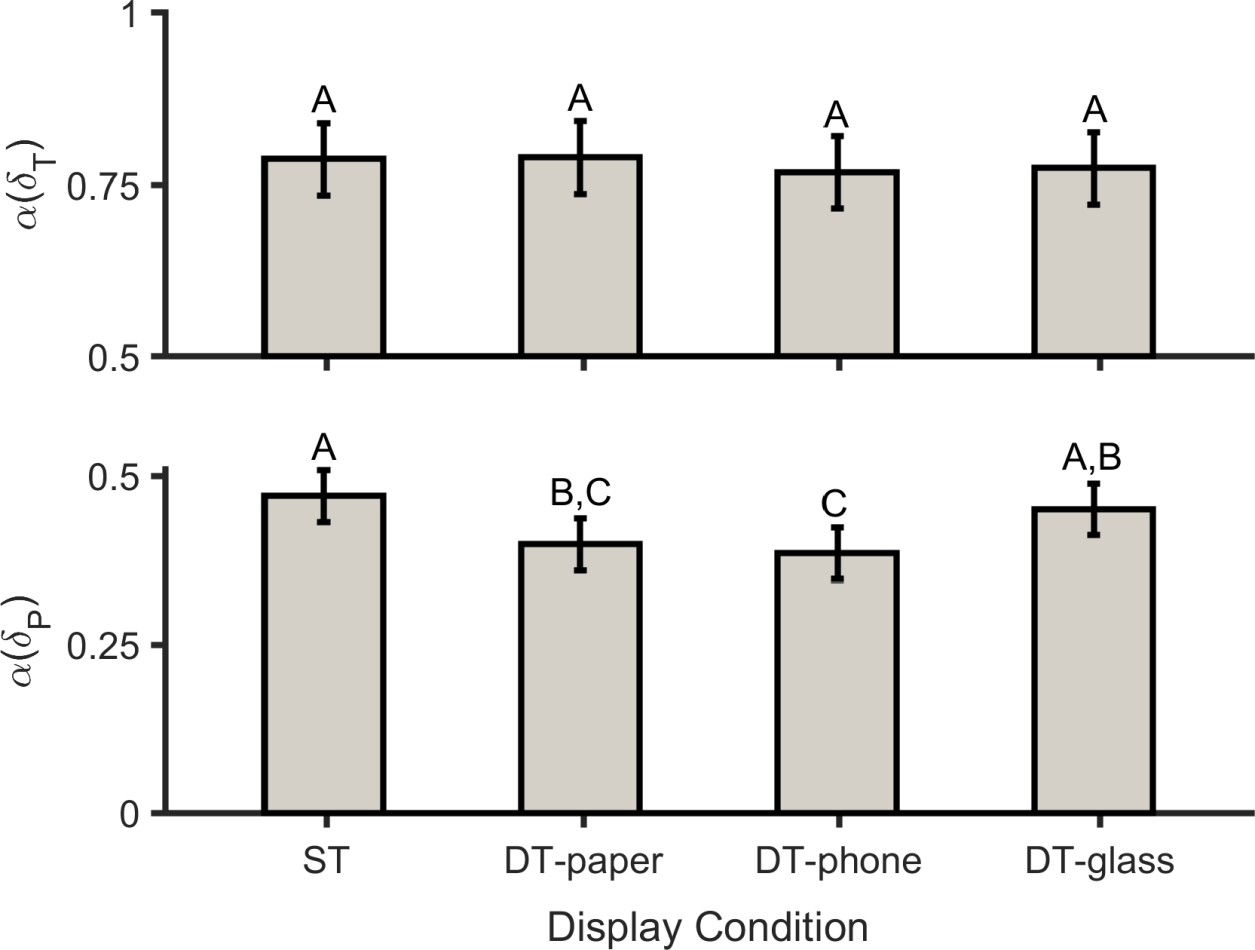
Temporal structure of variation in the GEM direction (*p* = 0.887 and *ηp2* = 0.012; top) and in the direction perpendicular to the GEM (*p* = <0.001 and *ηp2* = 0.269; bottom) for single-task walking (ST), and for dual-task walking while using the paper-based system (DT-paper), the smartphone (DT-phone), and the smart glasses (DT-glass). The number of participants was 20. Values in conditions not sharing same letters are significantly different.

The effect of DC on the magnitude of MV in the GEM direction was of medium size, and approached significance, while the DC effect was significant on the temporal structure of variability in the direction perpendicular to the GEM (Table 5). In all display conditions, the magnitude of variability in the GEM direction (*σ*(*δ*_*T*_) > 1.0) was higher than in the non-relevant GEM direction (*σ*(*δ*_*P*_) < 1.0; Fig 6). In general, though only approaching significance, participants exhibited higher variations in the GEM direction in the DT-paper (*p* = 0.057) condition compared to the ST condition, while these variations slightly increased in the DT-phone (*p* = 0.197) and DT-glass (*p* = 0.374) conditions (Fig 6, top). Based on DFA analysis, the temporal structure of variability in all of the display conditions was persistent (*α*(*δ*_*T*_) > 0.5) in the GEM direction, and uncorrelated (*α*(*δ*_*P*_) ≈ 0.5) or anti-persistent (*α*(*δ*_*P*_) < 0.5) in the direction perpendicular to the GEM (Fig 7). Values of *α*(*δ*_*P*_) in the DT-paper and DT-glass conditions were significantly lower than in ST walking (*p* = 0.003 and *p* = <0.001, respectively). In contrast, *α*(*δ*_*P*_) values when using the DT-glass did not change significantly from the ST condition (*p* = 0.686; Fig 6 bottom). While the interaction effect of DC×G on *α*(*δ*_*T*_) approached significance, the effects of DC was relatively consistent for both genders.

**Table 5:** Summary of ANOVA results related to the GEM-based outcomes for 20 participants. Both *p-*values and effect sizes (*η*_*p*_^*2*^) are given for the main and interaction effects of different display conditions (DC) and gender (G) for SD of stride length, stride time, and stride speed. Significant effects are highlighted using bold font and effects approaching significance are italicized.

		DC	G	DC×G
*σ*(*δ*_*T*_)	*p(η*_*p*_^*2*^*)*	*0*.*071 (0*.*121)*	0.117 (0.186)	0.415 (0.051)
*σ*(*δ*_*P*_)	*p(η*_*p*_^*2*^*)*	0.108 (0.105)	0.110 (0.192)	0.402 (0.052)
*α*(*δ*_*T*_)	*p(η*_*p*_^*2*^*)*	0.887 (0.012)	0.486 (0.023)	0.084 (0.114)
*α*(*δ*_*P*_)	*p(η*_*p*_^*2*^*)*	**<0.001 (0.269)**	0.631 (0.013)	0.962 (0.005)

### Cognitive performance

**Fig 8:**
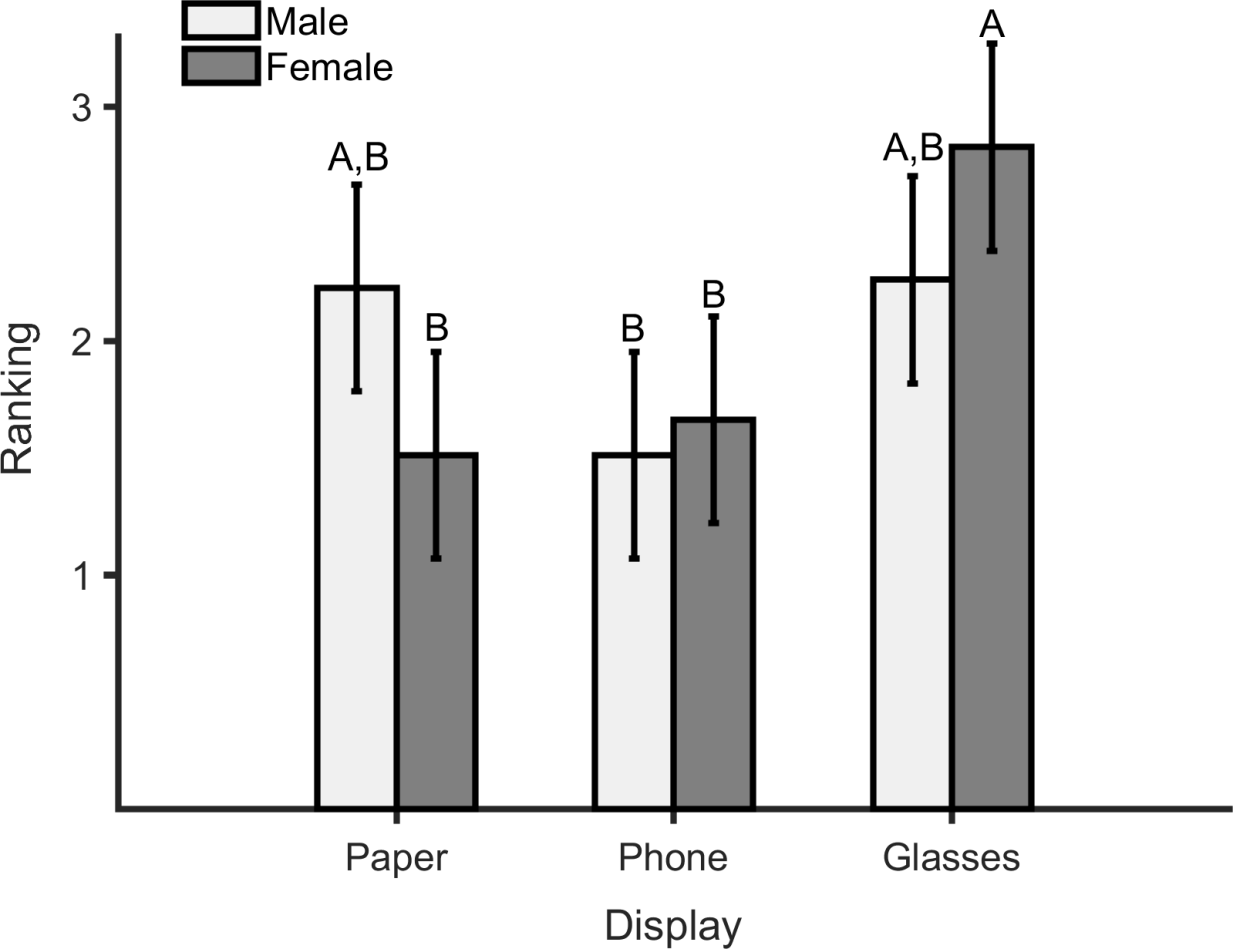
Mean preferences (rankings) of male and female participants for using different types of information display. 1 = first preference, 2 = second preference, and 3 = least preference.

There were significant main effects of display type on all responses and performance measures except for NASA-TLX scores (Table 6). Specifically, ease-of-use scores were higher in the smartphone condition vs. when using the smart glasses, and the smart glasses were perceived as less comfortable than both the paper-based system and smartphone condition (Table 7). Further, participants performed best on the Stroop test using the paper-based system; categorization performance was highest when using the smartphone and lowest when using the smart glasses; and performance in the arithmetic task was best when using the smartphone or smart glasses (Table 7). Rankings were significantly better for the paper-based system and smartphone, and, while not significant, the smartphone was overall preferred more often than the paper-based condition. Rankings were also significantly affected by a task condition x gender interaction. Females preferred the smartphone and paper-based system equally, ranking both displays as the most preferred device to use while walking (Fig 8). Males, in contrast, preferred the smartphone more than the paper-based system or the smart glasses, ranking the smartphone as the most preferred device and the latter two equally as the least preferred.

**Table 6:** Summary of ANOVA results related to the cognitive load outcomes for 20 participants. Both *p* values and effect sizes (*η*_*p*_^*2*^) are given for the main and interaction effects of different displays conditions (D) and gender (G) for questionnaire responses and task performance. Significant effects are highlighted using bold font, and effects approaching significant are italicized.

		D	G	D×G
NASA-TLX Overall workload	*p(η*_*p*_^*2*^*)*	0.232 (0.078)	0.548 (0.104)	0.630 (0.025)
Ease of Use	*p(η*_*p*_^*2*^*)*	**0.005 (0.249)**	0.447 (0.054)	0.182 (0.105)
Discomfort	*p(η*_*p*_^*2*^*)*	**<.001 (0.586)**	**0.044 (0.465)**	**0.035 (0.181)**
Participants performance (Stroop test)	*p(η*_*p*_^*2*^*)*	**0.001 (0.283)**	*0*.*056 (0*.*792)*	0.313 (0.019)
Participants performance (categorizing task)	*p(η*_*p*_^*2*^*)*	**0.021 (0.193)**	**0.041 (0.445)**	0.627 (0.0260)
Participants performance (arithmetic task)	*p(η*_*p*_^*2*^*)*	**<0.001 (0.387)**	0.137 (0.666)	0.357 (0.055)
Ranking	*p(η*_*p*_^*2*^*)*	**<0.001 (0.278)**	1 (0)	**0.019 (0.141)**

**Table 7:** Summary of results of pairwise comparisons related to cognitive load outcomes for 20 participants. Both least-square means (LMS) and 95% confidence intervals (CI) are given for the different displays, for questionnaire responses and task performance. Values in a row not sharing same superscripted letters are significantly different.

		Paper-based system	smartphone	Smart Glasses
NASA-TLX Overall workload (0–100)	LMS	52.29	54.58	56.00
	(CI)	(45.73–58.85)^a^	(48.02–61.14)^a^	(49.44–62.56)^a^
Ease of Use (1–5)	LMS	3.70	4.06	3.28
	(CI)	(3.28–4.07)^ab^	(3.68–4.40)^a^	(2.81–3.68)^b^
Discomfort (1–5)	LMS	1.31	1.38	1.83
	(CI)	(1.19–1.47)^b^	(1.24–1.55)^b^	(1.60–2.13)^a^
Stroop test (% completed)	LMS	78.56	76.28	73.93
	(CI)	(72.31–84.78)^a^	(70.04–82.52)^ab^	(67.7–80.17)^b^
Categorizing task (% completed)	LMS	86.67	88.33	83.54
	(CI)	(82.77–90.56)^ab^	(84.43–92.23)^a^	(79.64–87.43)^b^
Arithmetic task (% completed)	LMS	71.22	75.88	78.36
	(CI)	(63.13–78.48)^b^	(68.34–82.73)^a^	(71.08–85.01)^a^
Ranking (1–3)	LMS	1.869	1.587	2.545
	(CI)	(1.558–2.180)^b^	(1.276–1.897)^b^	(2.234–2.855)^a^

Open-ended question responses were compiled and categorized into several consistent categories–simplicity, usefulness, and comfort–and were subsequently labeled as positive or negative (summarized in Table 8). Participants appeared to prefer the smartphone condition overall, and the smart glass condition the least. More specifically, the smartphone condition was found to be the simplest condition, while participants considered the smart glasses to be the least simple condition. The most and least comfortable conditions were the paper-based display and smart glasses, respectively. Regarding usefulness, responses were divided evenly, with the glasses condition considered as both the most and least useful.

**Table 8:** Frequency of open-ended question responses categorized by simplicity, usefulness, and comfort for each display type.

		Number of Responses								
Category	Response Summary	Paper-based system			Smartphone			Smart Glasses		
		Total	Female	Male	Total	Female	Male	Total	Female	Male
Simplicity (Positive)	Images were clear and easy to see; Display was easy to use while walking	8	4	4	11	6	5	4	2	2
Simplicity (Negative)	Display was difficult to use while walking	6	3	3	4	2	2	7	4	3
Usefulness (Positive)	Display was lightweight, depth adjustable, and efficient; No glare	11	4	7	11	5	6	14	5	9
Usefulness (Negative)	Display was not stable and distracting	13	5	8	10	5	5	14	8	6
Comfort (Positive)	Display allowed for more control while walking; No eye strain	7	5	2	3	2	1	4	3	1
Comfort (Negative)	Display caused discomfort, instability, and blurred vision	7	4	3	6	2	4	13	7	6

## Discussion

Our primary goal in this study was to investigate the effects of different types of information display on gait variability. Our results supported the first hypothesis. Specifically, several measures derived from gait parameters were affected by the addition of cognitive tasks. Participants showed higher stride time variability (STV) when they used an information display compared to the single-task condition (while not statistically significant for all conditions; Fig 4, top). Similar to our results, in several earlier studies STV was also observed to increase when participants performed attentional cognitive tasks during walking [60–62]. A significantly higher STV in the DT-paper condition, relative to the other conditions, also suggests that it was the most demanding. One explanation for this is that during the DT-paper condition participants had to flip the pages manually; thus, participants had to allocate more cognitive resources for this condition. Since STV and the risk of a fall are associated [63], there is the implication that such a risk was elevated in all the current dual-task conditions (and especially in the DT-paper condition). In addition, stride speed variability decreased in all three dual-task conditions, with a substantial reduction in the DT-glass condition (Fig 4, bottom), due to increases in STV and decreased in stride length variability. Maki [64] found that increases in stride speed variability were directly related to falling risk, so our results regarding stride speed variability may imply a decreased risk of falls in dual-task conditions. Furthermore, a sole emphasis on variability in gait parameters (i.e., stride length variability, stride time variability, and stride speed variability), and limitations of linear methods in detecting MV patterns [33], may provide inconsistent evidence regarding fall risk.

Nonlinear analysis (i.e., SaEn), however, revealed clearer evidence regarding walking patterns in the different conditions. Higher values of SaEn indicate higher complexity and lower regularity in the time series [55]. In this study, participants exhibited more erratic walking in the DT-paper and DT-phone conditions compared to ST, as evident in a loss of complexity in their stride time [54]. However, SaEn values for DT-glass and ST were similar. This information regarding SaEn (stride time) may suggest that using the paper system and phone imposed a higher risk of falls compared to the ST condition. SaEn, however, measures walking variability for each gait parameter separately. GEM-based analyses, in contrast, use all stepping variables simultaneously to quantify both the magnitude and structure of gait variability [44]. As such, GEM-based outcomes potentially provide additional information on how the CNS regulates movement in the dual-task conditions. For all three display conditions, participants tended to have higher MV in the GEM direction compared to the control (single task) condition (while not statistically significant), which suggests that they employed the benefits of abundant solutions [65] to adapt to increasing attentional demands. Our results, however, are inconsistent with a previous report [18], in which healthy young people performed the Boston Naming Test during walking. In this earlier study, participants had lower variations in the GEM direction and higher variations in the direction perpendicular to the GEM, compared to the ST condition. Previous studies, though, have found that the effects of different types of cognitive task on gait parameters were inconsistent [60, 66], and that such differences may stem from each task requiring different attentional resources [67]. Therefore, a potential explanation for the inconsistency between our results and the study of Decker et al. [18] is that our use of a set of cognitive tasks was clearly different from their use of a single test. Based on the GEM-based analysis results, however, we conclude that the CNS adapts to dual-task conditions by manipulating the structure of variability (i.e., *σ*(*δ*_*T*_) and *σ*(*δ*_*P*_)). It is worth mentioning that we might have observed more substantial and statistically significant increases in the magnitude of MV in the GEM direction if the cognitive tasks were more challenging. Future work should thus be conducted to investigate the effects of task difficulty on the extent of MV.

The three display conditions here had different effects on the temporal structure of MV. Results from DFA analysis in the non-GEM-relevant direction compared to GEM direction showed that participants corrected their movements in the former direction more tightly [44]. Similar to previous studies [43, 44], *α*(*δ*_*P*_) in the ST condition was uncorrelated or slightly anti-persistent. This pattern was similar in the DT-glass condition. However, participants appeared to regulate their walking variability in the non-GEM-relevant direction more frequently in the DT-paper and DT-phone conditions, in contrast to the ST condition (*α*(*δ*_*P*_) << 0.5; Fig 7, bottom). This behavior indicates that the CNS stabilized walking steps in the DT-paper and DT-phone conditions to a greater extent than in the ST and DT-glass conditions, by maintaining GEM-based strategies more strictly [18]. Considering the temporal structure of variations in the non-GEM relevant direction, and SaEn results for stride time, we conclude that gait adaptability in the both DT-paper and DT-phone conditions (i.e., “head-down” conditions) was worse than the baseline. In the DT-paper and DT-phone conditions, participants held their heads down (Fig 3, middle), while in the latter two conditions they had to hold their heads up (Fig 3, right and left). To our knowledge, no prior studies have compared the effects of head-up vs. head-down postures on the variability of gait parameters. Further investigation is required, though, to determine whether the differences in temporal structure of variability between display conditions are due to the type of information display or due to the differences in head posture. However, our results are consistent with previous findings in the context of dual-task driving; several studies have found that head-up driving tasks impair driving performance to a lesser extent than head-down tasks [16, 68, 69].

We also evaluated different information displays from a cognitive perspective. Given the lack of substantial differences in NASA-TLX scores, the mental workload involved with each display was likely be similar, though display preference and task performance clearly varied. In general, participants most preferred the smartphone condition over either the paper-based system or the smart glasses. From responses to the open-ended question, participants noted that the smartphone was the easiest to control and most familiar, yet they did not like having to look down for a long period of time or the backlight. Since performance in the categorizing task was highest when using the smartphone, this suggests that a smartphone display is preferred for pictures (vs. reading words or numbers). Crowley et al. [70] showed that reading or texting on a smartphone significantly hinders walking performance and awareness. However, it is unknown whether altering the dual-task demands, perhaps involving looking at a picture, would have the same effect. As previously noted, different cognitive tasks may require different attentional resources [67]. Therefore, the high performance at categorization found here may be due to the type of attentional resources required for this task.

The second most preferred display was the paper-based system. From the responses obtained, participants considered this system to be the most comfortable, and noted that the smartphone backlight and the weight of the glasses were too uncomfortable. This is consistent with a previous report, in which participants experienced increased discomfort and eye strain when scanning text on a screen for both short and longer periods compared to scanning text on paper [71]. Further supporting these responses, Stroop test performance was higher when participants used the paper-based display, implying that tasks involving words or reading are better accomplished when using paper rather than when reading from a screen. Wright and Lickorish [72] found similar results; scanning text, speed, and accuracy all increased when a paper-based system was used rather than a screen display. Therefore, our results suggest that increased performance in the Stroop test is correlated with comfort rather than simplicity or usefulness. The paper-based system received poor reviews regarding simplicity and usefulness, likely due to participants needing to flip pages.

Finally, the least preferred display was the smart glasses. From the participant responses, this condition was evenly divided between most and least useful. The negative responses regarding usefulness were focused primarily on the structure of the device, rather than the display, specifically that the weight of the glasses was uncomfortable and hard to balance. Regarding display type specifically, the smart glasses were determined to be the most useful. Positive responses for usefulness suggested that participants liked the hands-free aspect of the device and the eye-level screen, which allowed participants to complete the tasks without looking down. This is consistent with a report by Liu and Wen [73], in which head-up displays allowed for significantly faster response times, more control, and caused less mental stress, in tasks that required a quick response. Arithmetic performance here was significantly higher when using both the smartphone and smart glasses condition, and highest when using the smart glasses display. An increased performance in the arithmetic task with the smart glasses may be due to the freedom to look up, in conjunction with the participant’s need for extended viewing and quick response time.

Based on the above discussion, cognitive analyses did not support the motor variability outcomes. In general, participants preferred to use the smartphone and paper-based system rather than the smart glasses. In contrast, gait adaptability was better for the smart glasses. One potential reason for the inconsistency between these results is that participants did not have adequate experience using smart glasses, which made it an uncomfortable device for them. Smart glasses are also not a well-developed technology; for example, Brusie et al. [74] evaluated two types of smart glasses from a usability perspective, and found that both were not sufficiently mature to satisfy users. There is some difficulty in interpreting our results, specifically in terms of separating the effects of the technology itself, as an information display, from usability aspects (e.g., comfort).

As we hypothesized, there were differences in the sensitivity of several measures of variability to the use of three types of information displays. Cycle-to-cycle SD of stride time and stride speed, SaEn of stride length, and the temporal structure of variability in the non-GEM-direction (*α*(*δ*_*P*_)) all had “large” effect sizes, which implies that these measures were highly sensitive to changes that occurred due to the different walking conditions. As discussed above, separately quantifying the magnitude of variability of gait parameters (stride length, stride time, and stride speed), using cycle-to-cycle SD, cannot reveal how the CNS might employ the benefits of MV for the different walking conditions. While the linear method (i.e., cycle-to-cycle SD) was sensitive to differences between walking conditions, we conclude that it should not be used alone for studying gait variability.

Values of SaEn (stride time) and *α*(*δ*_*P*_) had the highest effect sizes among all of the variables, and provided the clearest results. Alternatively, the phone condition had smaller effects on SaEn compared to the DT-paper, while we observed an inverse pattern for *α*(*δ*_*P*_). One possible reason for the divergence in these patterns is that SaEn only quantified variability for one gait parameter, while GEM analysis considered variabilities for all parameters simultaneously. Another possibility is that in the DT-paper and DT-phone conditions (i.e., the head-down conditions), participants could perceive small variations in their walking kinematics, and they allocated some of their cognitive resources to correct these variations. In the DT-paper condition, however, they had to flip the pages, limiting their resources for regulating their walking patterns. A similar reasoning can be employed for the SaEn Results analysis; the DT-paper condition was more demanding than the DT-phone, leading to higher SaEn values than the DT-paper.

It is worth noting that we calculated SaEn for different combinations of *r* and *m*. Derived values of the SaEn (stride time) were consistent for different *r* values, however the two other variables (i.e., SaEn (stride length) and SaEn (stride speed)) were very sensitive to *r*. Based on this inconsistency, we could not interpret the results for stride speed and stride length. Similar to Yent et al. [54], for *m* = 4 the algorithm diverged in most of the conditions and was excluded from our analysis. We did not find any statistical differences between SaEn obtained from time series using *m* = 2 and 3, but the results for *m* = 3 were more clear (i.e., we could easily compare single-task with dual-task conditions). Finally, some of the values of SaEn (stride length) and SaEn (stride speed) for *m* = 3 and small values for *r* did not converge, and we therefore chose *r* = 0.25. Based on this and previous suggestions [54], we encourage future investigators to explore different parameters to obtain the best results for SaEn analysis.

Our conclusions and recommendations, though, require some caution due to inherent study limitations. First, a relatively small number of participants were recruited. Second, there is the potential for different behaviors occurring during over-ground versus treadmill walking, though some recent evidence suggests that these differences may be minimal [75]. It remains unclear, though, regarding the extent to which the current results generalize to the use of different information displays generally, especially outside of a laboratory environment (e.g., in which diverse visual information is present in addition to the display). Third, each walking condition took five minutes; however, performing each of the cognitive tasks for five minutes was found, in pilot work, to be quite challenging (boring) for participants. We thus included all three cognitive tasks in each dual-task condition, but as a result could not investigate separately the effects of each cognitive task on MV and cognitive performance. Further investigation is thus needed to explore how each cognitive task might influence gait variability. Fourth, several outcomes for the DT-paper and DT-phone conditions significantly changed compared to the ST. However, differences between the DT-glass and head-down conditions (i.e., DT-paper and DT-phone) were sometimes less evident. We believe that the latter occurred because the total amount of cognitive load during the DT conditions consisted of the as follows cognitive load of the secondary tasks and display types. On the other hand, the cognitive tasks were similar for all of the DT conditions, and differences in cognitive load between two DT conditions were smaller than the differences between the DT and ST conditions. In this study, however, we were particularly interested in learning which of information displays might have the most adverse effects on gait performance. When we observed important differences between the MV outcomes (especially for the DFA and SaEn analysis) of one of the DT-conditions and the baseline, we concluded that walking performance was deteriorated in those DT-conditions and that this implies potential risks of falling. Fifth, though the participants had time to gain experience in using the smart glasses, unfamiliarity with using this new technology could have affected the outcomes. It also should be noted that several types of smart glasses are available in the market, and it would be interesting to explore how different types might influence outcomes related to gait variability. In addition, the participants tested here were healthy and young; however, not all potential users of HWDs fit this description. It may be possible, for example, to develop new HWD applications to help patients with different pathologies. Future work should also test the effects of HWDs on motor variability during gait with a more diverse group of individuals, such as with respect to health and age. Lastly, each information display required different levels of hand involvement. For example, the single-task condition allowed both arms to swing naturally, while in dual-task conditions the participant had to hold an object (i.e., paper, phone, and smart glasses’ remote). In addition, for the DT-paper condition, participants at times needed to use their second hand to flip pages. Magnani et al. [76] found that hand posture while using a cellphone during walking may increase attentional demand. Though we asked the participants to maintain a similar hand posture in all of the DT conditions, it was impossible to completely eliminate the effects of hand posture on the results due to the physical requirements of each information display.

In summary, gait performance was less affected by the smart glasses than when using either a paper-based or smartphone systems for information presentation, both of which required a head-down walking posture. We suggest that smart glasses are a promising technology for reducing the risk of an adverse gait event (e.g., a fall), but that this new technology may still not be matured sufficiently for implementation (e.g., into industrial environments). We also found that variability in the GEM direction can be an effective solution for the CNS to adapt to challenging walking conditions, such as those that occur in dual-task conditions. Furthermore, increasing MV can potentially be a useful tool for maintaining gait performance and decreasing the risk of a fall. Finally, we used different methods for quantifying MV, and our results suggest that the GEM analysis and SaEn for stride time can be a fruitful method for studying gait variability.

## Supporting information

S1 File Excel workspace including all needed cognitive results for statistical analyses. (XLSX)

S2 File Excel workspace including all needed motor variability results for statistical analyses. (XLSX)

## Abbreviations

MV: Motor variability
GEM: Goal Equivalent Manifold
PWS: Preferred Walking Speed
L_n_: Stride length
T_n_: Stride time
S_n_: Stride speed
SaEn: Sample entropy
δ_T_: Variations in the GEM direction
δ_P_: Variations in the direction perpendicular to the GEM
α: Scaling exponent
STV: Stride time variability
